# *Origanum Majoranum *Extract Modulates Gene Expression, Hepatic and Renal Changes in a Rat Model of Type 2 Diabetes

**Published:** 2016

**Authors:** Mohamed Mohamed Soliman, Mohamed Abdo Nassan, Tamer Ahmed Ismail

**Affiliations:** a *Department of Biochemistry, Faculty of Veterinary Medicine, Benha University, Egypt. *; b *Medical Laboratory Department, Faculty of Applied Medical Sciences, Turabah, Taif University, Saudi Arabia. *; c *Departments of Pathology and Physiology. *; d *Faculty of Veterinary Medicine, Zagazig University, Egypt.*

**Keywords:** Dyslipidemia, Gene expression, *Origanum* extract, Hepatic and renal histopathology, Type 2 diabetes

## Abstract

The present study was conducted to test the effect of *Origanum Majoranum* *Extract *(OME) of leaves on alterations induced in a model of type 2 diabetic rats. Adult male Wistar rats were fed high fat diet for 3 weeks and injected a single dose of streptozotocin (35 mg/kg) intraperitoneally to induce type 2 diabetic rats. Diabetic rats were given aqueous extract of OME in a dose of 20 mg/kg orally for 3 weeks. Changes in lipid profiles, glucose, insulin, expression of some genes related to glucose metabolism and histopathological changes in liver and kidney were examined. Administration of OME improved and normalized dyslipidemia recorded in type 2 diabetic rats together with reduction in glucose and insulin levels. OME induced up-regulation in gene expression of glucose [adiponectin and glucose transporter-2 (GLUT-2)] and lipid metabolism [lipoprotein lipase (LPL)]. Moreover, OME normalized histopathological changes occurred in liver and kidney of diabetic rats. OME decreased lipids accumulation in liver and kidney and increased regeneration of hepatic parenchyma and restored normal renal architecture with disappearance of fat droplets. In conclusion, OME improved dyslipidemia associated with type 2 diabetes through regulation of genes related to glucose and lipid metabolism.

## Introduction

Diabetes mellitus (DM) is a metabolic disease characterized by alterations in carbohydrate, protein, and lipid metabolism. It is caused either by defect in insulin action or secretion. Diabetes is mostly associated with an increased risk of coronary heart disease, stroke, hypertension, renal failure and dyslipidemia; all-cause mortality (1). The onset of hyperglycemia is often preceded by insulin resistance that causes adverse health effects. Alterations in lipid metabolism cause an increase in mobilization of stored lipids that elevate free fatty acids in the blood. Elevation of free fatty acids is the possible cause for the decrease in muscle glucose uptake, and increase in liver glucose production, that contribute to elevated blood glucose levels (2). The metabolic syndrome is caused by the increase in plasma levels of insulin and glucose resulting in insulin resistance. The incidence of insulin resistance is increased by feeding high fat, high calorie, and low-fiber diets (3, 4). It is generally accepted that high-fat diets alone for 3 months (5), or together for 3 weeks with medium dose of streptozotocin (6) can be used to generate a rodent model for the metabolic syndrome with insulin resistance and type 2 diabetes.

Type 2 diabetes and/or insulin resistance is a metabolic disorder characterized by high serum low density lipoprotein and blood cholesterol (dyslipidemia). Dietary factors such as continuous ingestion of high amounts of saturated fats and cholesterol are believed to be directly related to hypercholesterolemia and atherosclerosis (7). Hyperlipidemia is a major problem to many societies as well as health professionals because of the close correlation between cardiovascular diseases and lipid abnormalities (8). Adipose tissue is responsible for the secretion of variable signaling molecules called adipokines. Adipokines include free fatty acids, leptin, plasminogen activator inhibitor-1, resistin, TNF-α and adiponectin (9-14). The dysregulation of adipokines is incorporated in the incidence of obesity, type 2 diabetes, and cardiovascular diseases (15). Treatment of type 2 diabetes mellitus depends on oral hypoglycemic medications that contain peroxisome proliferator activated receptor gamma (PPAR-γ) and thiazolidinediones. These drugs induce improvement in hyperinsulinemia and dyslipidemia (16). Regular usage of many herbs has been recommended in the management of hyperlipidemia (17). Plant extracts are safer than chemical products and is more popular, since drugs of synthetic origin may have a negative impact on health state (18).

Marjoram or Oregano (*Origanum majoranum*; OM) is found all over Asia, Arabian peninsula, Africa, America and Europe (19). Oregano contains oleanolic and ursolic acids, flavonoids and hydroquinones, caffeic, rosemarinic, and lithospermic acids, tannins, and phenolic glycosides. Phenolic compounds of oregano represent 71% of the total oil. The polar phenols, thymol and carvacrol, are responsible for many of the properties of the essential oil (20-22). Its aromatic leaves are used as flavoring and other culinary purposes. The essential oil is widely used as antihyperglycemic (23), anti-inflammatory (24, 25), cytotoxic (26), antioxidant (27, 28), antifungal, antibacterial (30), antithrombin (31), and anticarcinogenic effects (32). Nowadays, most diabetic patients prefer the traditional plant-based medicines for their primary health care (33). Therefore, in this study we first investigate the effects of the aqueous extract of *Origanum majoranum *leaves on dyslipidemia, hypoglycemia and hyperinsulinemia induced in a rat model of type 2 diabetes. Then, we show the changes in hepatic gene expression together with the histopathological findings in liver and kidney.

## Experimental

*Materials*

Marjoram (*Origanum majoranum*) leaves were bought from Saudi Arabia markets and the plant was identified and authenticated by specialized agronomist. Qiazol reagent was from QIAGEN Inc., Valencia, CA. Oligonucleotide PCR primers were from Macrogen Company, GAsa-dong, Geumcheon-gu. Korea. Streptozotocin (STZ), was purchased from sigma Aldrich, USA. Reverse transcriptase was from SibEnzyme Ltd. Ak, Novosibirsk, Russia. Solvents and related materials were from ADWIA pharmaceutical company, Egypt.

*Preparation of Origanum majoranum extract *(OME)

The marjoram extract was prepared according to studies of Benavides *et al*. 2010 (34). In short, three hundred grams of *Origanum majoranum* leaves were minced in water, boiled in distilled water (1.5 liter) at 60 °C for 15 minutes, and then filtered to obtain the aqueous extract. The Aqueous extract was dried at 60 ºC in a stove for 24 hours. After filtration, a sample was separated for determination of the solid concentration.

*Experimental design*

Male Wistar rats (30 rats), 6 weeks old, weighting 110-170 g, were selected randomly. Rats were exposed to 12 h/12 h day light cycle with free access to food and water. Rats were divided into 3 groups (10 rats per group). Control group was fed normal diet, and the remaining 20 rats were fed high fat diet (HFD) for 3 weeks. The HFD constitutes 15.5% protein, 38.8 % fat and 45.7% carbohydrates, by calories. Induction of type 2 diabetes in rats was based on the protocol stated by Srinivasan and his colleagues (6) by intraperitoneal injection of medium dose of STZ (35 mg/kg BW) together with HFD. Diabetes was confirmed by hyperlipidemia, hyperglycemia and hyperinsulinemia (Table 2). Diabetic rats (n=20) were subdivided into 2 subgroups, diabetic group (n=10), Diabetic plus *Origanum* (20 mg/kg/day) for 2 weeks. Dose of Origanum was used based on studies of Lemhadri *et al*. (23) and Mohamed and Nassier (35). At the end of experiments, all rats were decapitated after overnight fasting and blood was collected for serum extraction. Liver, epididymal adipose tissue and kidney tissues were preserved either in Qiazol for RNA extraction and gene expression (liver and adipose tissue) or formalin for histopathology (liver and kidney).

*Biochemical Measurements*

Glucose, insulin, total triglycerides (TG), total cholesterol, low and very density lipoproteins (LDL, VLDL) and high density lipoproteins (HDL) were measured using commercial enzymatic and colorimetric kits. Kits were from BIODIAGNOSTIC company, Dokki, Giza, Egypt.

*RNA extraction, cDNA synthesis and Semi-quantitative RT-PCR analysis*

For preparation of total RNA, epididymal adipose tissue and liver (approximately 100 mg per sample) were collected from animals, flash frozen in liquid nitrogen, subsequently stored at -70 °C in 1 mL Qiazol (QIAGEN Inc., Valencia, CA). Frozen samples were homogenized using Polytron 300 D homogenizer (Brinkman Instruments, Westbury, NY). To homogenate, 0.3 mL chloroform was added. The mixtures were shaken for 30 seconds then centrifuged at 4 °C and 12,500 rpm for 15 min. The supernatant aqueous clear layer was transferred to new set of tubes, and an equal volume of isopropyl alcohol was added to the samples, shacked for 15 seconds and centrifuged at 4 °C and 12,500 rpm for 15 min. The RNA pellets were washed with 70% ethanol, briefly dried up then, dissolved in DEPC water. The prepared RNA integrity was checked by electrophoresis. RNA concentration, integrity and purity were determined spectrophotometrically at 260 nm. For synthesis of cDNA, 1 µg of total RNA and 0.5 ng oligo dT primer in a total volume of 11 µL sterilized DEPC water was incubated in the PeX 0.5 thermal Cycler (Thermo Electronic Corporation, Milford, Ma) at 65 °C for 10 min for denaturation. Then, 4 µL of 5X RT-buffer, 2 µL of 10 mM dNTPs and 100 U Moloney Murine Leukemia Virus (M-MuLV) Reverse Transcriptase (SibEnzyme Ltd. Ak, Novosibirsk, Russia) were added and the total volume was completed up to 20 µL by DEPC water. The mixture was then re-incubated in the thermal Cycler at 37 °C for one hour, then at 90 °C for 10 min to inactivate enzyme. Specific primers for examined genes (Table 1) were designed using Oligo-4 computer program and were synthesized by Macrogen (Macrogen Company, GAsa-dong, Geumcheon-gu. Korea). PCR was conducted in a final volume of 25 µL consisting of 1 µL cDNA, 1 µL of 10  picomolar (pM) of each primer (forward and reverse), and 12.5 µL PCR master mix (Promega Corporation, Madison, WI) the volume was brought up to 25 using sterilized, deionized water. PCR was carried out using a PeX 0.5 thermal Cycler with the cycle sequence at 94 °C for 5 minutes one cycle, followed by different cycles according to each gene; each cycle is consisted of denaturation at 94 °C for one minute, annealing at the specific temperature corresponding to each primer (Table 1) and extension at 72 °C for 1 minute with additional final extension at 72 °C for 5 minutes. Expression of G3PDH mRNA as a house keeping gene was examined as a reference detected by using specific primers (Table 1). PCR products were electrophorized on 1.5% agarose (Bio Basic INC. Konrad Cres, Markham Ontario) gel stained with ethidium bromide in TBE buffer (Tris-Borate-EDTA). PCR products were visualized under UV light and photographed using gel documentation system. The intensities of the bands were quantified densitometrically using ImageJ software (http://imagej.en.softonic.com/). 

**Table.1:** PCR conditions of variable gene expressions in adipose tissue and liver

**mRNA expression**	**Forward primer**	**Reverse primer**	**PCR cycles and annealing conditions**
**PEPCK** **(239 bp)**	5‘-TTTACTGGGAAGGCATCGAT- 3'	5-TCGTAGACAAGGGGGCAC-3'	30 cycles, 52°C for 1 min
**GLUT-2** **(330 bp)**	5'-AAGGATCAAAGCCATGTTGG-3'	5'-GGAGACCTTCTGCTCAGTGG-3'	30 cycles, 55°C 1 min
**LPL (269 bp)**	5'-CCTGATGACGCTGATTTTGT-3'	5’-AGGCAAGCTGGTGAGGATCTG-3'	24 cycles, 60°C for 45 sec
**Leptin (244bp)**	5-'CCTGTGGCTTTGGTCCTATCTG-3’	5'-TATGCTTTGCTGGGGTTTTC-3'	35 cycles, 61°C for 1 min
**Adiponectin (500 bp)**	5'-CTCCACCCAAGGAAACTTGT-3'	5'-CTGGTCCACATTTTTTTCCT-3'	35 cycles, 59°C for 1 min
**PPAR-(550 bp)**	5’-CATTTCTGCTCCACACTATGAA-3'	5'-CGGGAAGGACTTTATGTATGAG-3'	35 cycles 51°C for 1 min
**GAPDH** **(309 bp)**	5'-AGATCCACAACGGATACATT-3'	5-TCCCTCAAGATTGTCAGCAA-3’	25 cycles, 52 °C 1 min

*Histopathological examination*

The collected specimens of liver form the sacrificed rats were fixed in 10% buffered neutral formalin solution for at least 24 hrs and then routinely processed. Paraffin sections of 5 micron thickness were prepared, stained with Hematoxylin and eosin stain (H&E) and then examined microscopically.

*Statistical Analysis*

Results are expressed as means ± S.E. for 5 different rats per each group. Statistical analysis was done using ANOVA and Fischer’s post hoc test, with p < 0.05 being considered as statistically significant.

## Results

*OME improves dyslipidemia associated with type 2 diabetes*

To test the effect of OME on T2DM, we administered orally *Origanum* in a dose of 20 mg/kg orally for 2 weeks. As seen in Table 2, there was an increase in all tested profiles. Administration of OME induced significant decrease in all dyslipidemia associated parameters (Table 2). OME decreased significantly (p<0.05) the elevation in glucose and insulin levels. Moreover, OME induced significant decrease in cholesterol, TG, LDL, VLDL and an increase in HDL levels and improved cholesterol ratio from 8.07% to 4.5%.

**Table 2:** Effect of Origanium extract on dyslipidemia induced in type 2 diabetic Wistar rats.

	**Insulin resistance**	**Control**	**Diabetic + Origanum**
Glucose (mg/dL)	178.6± 6.1*	93.3± 3.8	123.3± 32.01#
Insulin (IU/L)	7.3± 0.28*	2.3± 0.4	4.02± 0.15#
Cholesterol (mg/dL)	190.6± 4.4*	118.7± 2.9	145± 12.2#
Triglacylglycerols (mg/dL)	195.3± 10.5*	98.3± 6	150± 13.2#
LDL (mg/dL)	128± 9.6*	64.4.6± 4.4	84.7± 4.35
VLDL (mg/dL)	38.9± 2.1*	19.6± 2.2	30.3± 1.8
HDL (mg/dL)	23.6± 2.1*	34± 1.01	30.6± 0.9
Cholesterol ratio (TC÷HDL)	8.07%*	3.49%	4.8%#

*Data are presented as means ± S.E. S.E for 5different rats per each group.*

* *p<0.05 Vs control and *

# *p<0.05 Vs diabetic rats*

*Modulation of epididymal gene expression by OME administration*

**Figure 1:**
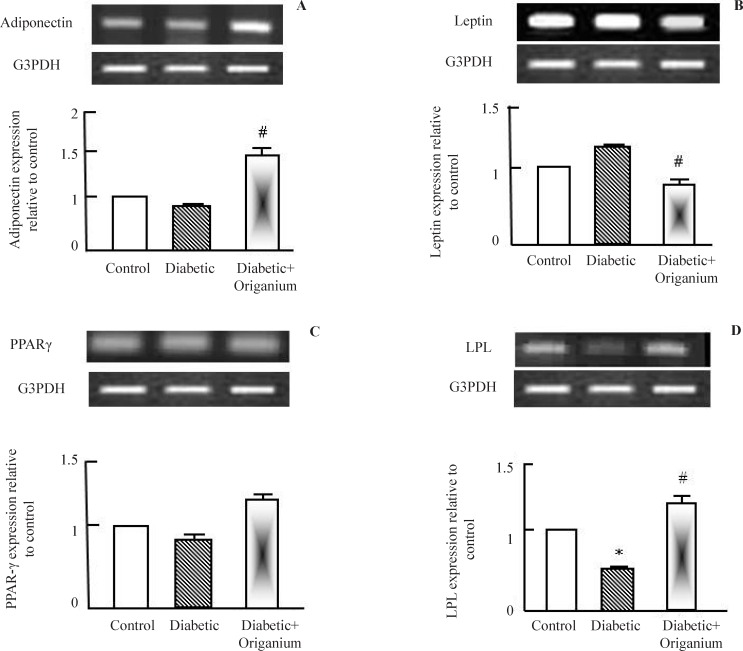
RT-PCR analysis of adiponectin, leptin. PARAR-γ and LPL expression after *Origanum* extract administration to type 2 diabetic Wistar rats for 2 weeks. RNA was extracted and reverse transcribed (2 μg) and RT-PCR analysis was carried for adiponectin, leptin. PARAR-γ and LPL genes. Densitometric analysis was carried for results from 5 different rats. *p<0.05 Vs control while # p<0.05 Vs diabetic group

**Figure 2:**
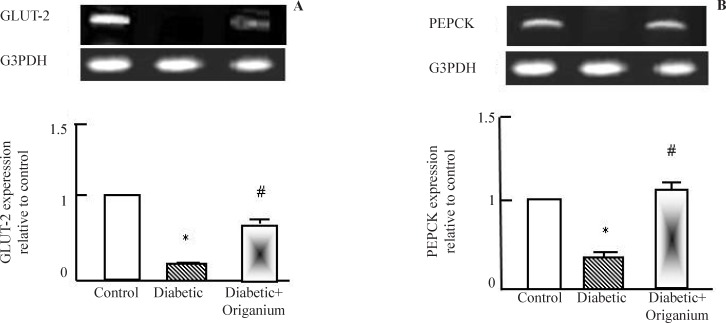
RT-PCR analysis of GLUT-2 and PEPCK expression after *Origanum* extract administration to type 2 diabetic Wistar rats for 2 weeks. RNA was extracted and reverse transcribed (2 μg) and RT-PCR analysis was carried for adiponectin, leptin. PARAR-γ and LPL genes. Densitometric analysis was carried for results from 5 different rats. *p<0.05 Vs control while # p<0.05 Vs diabetic group

To explore the molecular mechanisms by which OME improved dyslipidemia in type 2 diabetic rats, expression of adiponectin, leptin, PPAR-γ and LPL were examined. Adiponectin is insulin sensitizing protein secreted from adipose tissue to increase peripheral glucose utilization in liver and muscle. Adiponectin expression was decreased in type 2 diabetic rats and increased in *Origanum* administered rats (Figure 1 a). Next, we tested leptin expression, OME administration to diabetic rats decreased leptin expression (Figure 2b). We examined the expression of some lipid metabolism genes such as PPAR-γ and LPL. OME did not alter PPAR-γ expression (Figure 1.c). LPL expression was decreased in type 2 diabetic rats. OME administration for 2 weeks normalized and increased LPL expression confirming its role as a lipolytic herbal extract (Figure 1d).

*Hepatic expression of GLUT-2 and PEPCK expression after Origanum administration*

As known, T2DM is characterized by alteration in glucose metabolism. As seen in Figure 2 a and b, diabetes induced down-regulation in glucose transcporter-2 (GLUT-2) and phosphoenol pyruvate carboxykinase (PEPCK). Administration of OME induced significant normalization in GLUT-2 and PEPCK expression.

*Hepatic and renal histopathological findings*

**Figure 3:**
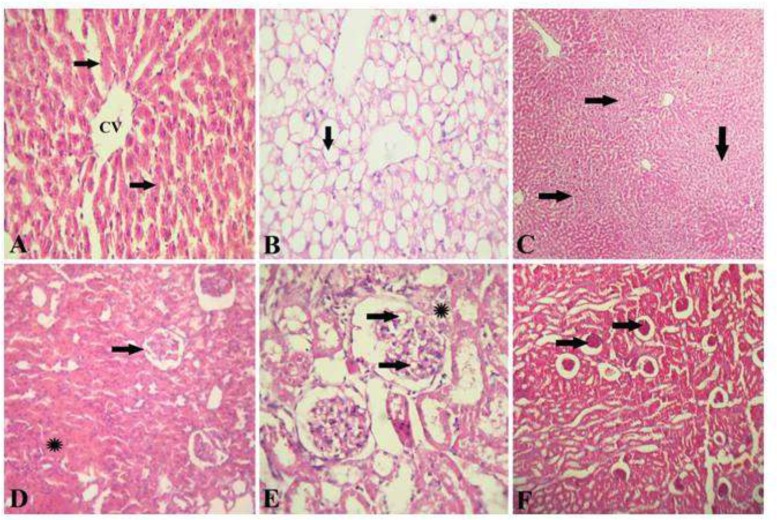
Hepatic and renal histopathology after *Origanum* extract administration. A, Photomicrograph of the liver of healthy control rat stained with H&E showing a normal hepatic architecture represented by hepatic lobule with a thin walled central vein (CV), hepatic cords (arrows) radiating towards the periphery alternating with hepatic sinusoids. (H&E x 300). B, photomicrograph of the liver of diabetic rats stained with H&E showed a signet-ring appearance of hepatocytes due to massive accumulation of fat extensively replacing the hepatic cytoplasm (arrow), or appearing as multiple small fat droplets (*). (H&E x 300). C, photomicrograph of the liver of diabetic rats treated with OM stained with H&E showing restoration of normal hepatic architecture with disappearance of fat droplets from hepatocytes cytoplasm and regeneration of hepatic parenchyma (H&E x 150). D, photomicrograph of the kidney of healthy control rat stained with H&E showed a normal renal architecture represented by normal glomeruli (arrow) and tubules (*). (H&E x 300). E, photomicrograph of the kidney of diabetic rats showing foamy lipid-containing mesangial cells expanding the glomerulus (arrows), the Bowman's capsule is moderately thickened forming synechia (*). (H&E x 1500). F, photomicrograph of the kidney of diabetic rat treated with OM stained with H&E showing restoration of normal renal architecture with disappearance of fat droplets from the glomeruli (arrows) (H&E x 150

The liver of healthy control rat showed normal hepatic architecture that represented by hepatic lobule with a thin wall central vein (CV), hepatic cords radiation toward the periphery alternated with hepatic sinusoids (Figure 3a). The liver of diabetic rats showed a signet ring appearance of hepatocytes due to massive accumulation of fat, extensively replacing the hepatic cytoplasm or appearing as multiple small fat droplets (Figure 3b). The liver of diabetic rats given OME showed restoration of normal hepatic architecture with disappearance of fat droplets from hepatocytes cytoplasm and regeneration of hepatic parenchyma (Figure 3c). The kidney of healthy control rat showed a normal renal architecture represented by normal glomeruli and tubules (Figure 3d). Kidney of diabetic rats showed foamy lipid-containing mesangial cells expanding the glomerulus, the Bowman's capsule is moderately thickened forming synechia (Figure 3e). The kidney of diabetic rats given OME showed restoration of normal renal architecture with disappearance of fat droplets from the glomeruli (Figure 3f).

## Discussion

In this study, we identified the recovery effects of OME on alterations induced during insulin resistance. OME normalized the changes occurred in T2DM and improved the expression of genes related to glucose metabolism (adiponectin and GLUT-2), also it normalized PEPCK expression. In addition, we demonstrated that OME induced restoration of normal hepatic and renal architecture with decrease in hepatic lipid accumulation. In fact, marketed preparations of oral hypoglycemic agents exhibit several side effects. Thus, there is a need for more effective and safe oral anti-hyperglycemic agents without any adverse effect, particularly those that normalize both insulin and glucose levels. Therefore, we used Origanum to examine its role in T2DM treatment.

Glycogen is the primary intracellular storable form of glucose and its level in various tissues especially skeletal muscle are a direct reflection of insulin activity as insulin promotes intracellular glycogen deposition by stimulating glycogen synthase and inhibiting glycogen phosphorylase (36). Moreover, thiazolidinediones, selective ligands for PPAR-γ, are critical for adipogenesis and act as insulin sensitizers, and regulates the transcription of its target genes such as aP2, LPL, leptin and fatty acid synthase (36, 37). Although the biologically relevant endogenous PPARγ ligands are unknown, exogenous ligands for PPARγ including free fatty acids (FFAs), prostanoids and the synthetic high-affinity anti-diabetic agents are well established. Plasma FFAs, elevated in obesity, can activate PPARγ through direct interaction with the ligand-binding domain of this receptor (38). Our findings reported that OME partially affect PPAR-γ expression and its action is dependent on lipolysis through its action on LPL expression but not on lipogenesis. Insulin integrates hepatic carbohydrate metabolism by increasing the biosynthesis of enzymes of glycolysis, glycogenesis, and pentose oxidative pathway; and by inhibiting gluconeogenesis (36). However, in our study because of insulin resistance and hyperinsulinemia, cells are not able to consume glucose under the effect of insulin but cells increased its internal gluconeogenesis under effect of Origanum. So, the normalization of PEPCK found in our study is a compensatory mechanism for hepatocytes to synthesize its internal energy source. Parallel with PK and PEPCK, is the pattern of GLUT2 expression. GLUT2 is a trans-membrane carrier protein that enables passive glucose movement across cell membranes. It is the main transporter for glucose between liver and blood and for renal glucose reabsorption (39). GLUT-2 expression is decreased in type 2 diabetic rats and administration of OME partially normalized it to improve hepatic glucose utilization.

Under the effect of Origanum, alterations in hepatic and renal cell function occurred as explained in histopathological findings and its administration normalized the expression of some genes related to carbohydrate and lipid metabolism such as adiponectin, GLUT-2 and LPL. Similar findings were reported for other herbal medications and drugs such as cinnamon and amylin (40, 41). This lowering effect of Origanum on triglycerides may be due to its action on fatty acids synthase activity in hepatocytes (42). It is well known that incidence of type 2 diabetes is accompanied by reduction in the activity and amount of several key enzymes as glucokinase, phosphofructokinase, and pyruvate kinase (43). Therefore, impairment in peripheral glucose utilization in type 2 diabetes and augmented cellular hepatic glucose production from gluconeogenesis occurred and that is in agreement with our findings. The glucose and insulin lowering effect of *Origanum* extract may be due to alteration of genes related to hepatic glucose production (44) and/or stimulation of glucose utilization by peripheral tissues by stimulating the expression of adiponectin and GLUT-2 in liver and peripheral tissues (40, 45). In addition, treatment of type 2 diabetes associated with insulin resistance by Origanum and normalization of blood glucose levels may be results from restoration of normal insulin sensitivity (23, 46).

As known, *origanum* leaves are currently used for the treatment of both type 1 and type 2 diabetes mellitus (23, 35, 44). The dose of *Origanum* used (20 mg/kg) is effective and the duration of treatment (2 weeks) were sufficient to normalize blood glucose levels in severely diabetic rats. After repeated oral administration, the glucose lowering activity of aqueous *Origanum* extract (20 mg/kg) was comparable to metformin treatment (45). We can report that the anti-hyperglycaemic effect of *Origanum* is very potent and cumulative. In addition, the plant extract could acts as an inhibitor of renal tubular glucose reabsorption (47). The main known constituents of organium have been demonstrated to be volatile compounds such as linalool, alcohols, phenols and terpens (48, 49). Oregano oils include carvacrol and thymol, are used as a dietary supplement for combating infections and relieving digestive and skin-related problems (50). All potentially act as antibacterial and antioxidants (51, 52). Additionally, flavonoids and/or polyphenols are active principles in many medicinal plants and natural products, and have beneficial effects for human health (53). These natural compounds could act separately or synergistically to cause the hypoglycemic effect of *Origanum*. So our findings support the concept of *Origanum* usage for treatment of diabetes. 

## Conclusion

In conclusion, *Origanum *extract is a useful herbal medication for treatment of type 2 diabetes. Moreover, *Origanum* improves insulin resistance and regulates gene expression of lipid and carbohydrate metabolism together with restoration of normal of hepatic and renal tissues architectures.
